# The oral-gut microbiota relationship in healthy humans: identifying shared bacteria between environments and age groups

**DOI:** 10.3389/fmicb.2024.1475159

**Published:** 2024-10-24

**Authors:** Carolina F. F. A. Costa, Teresa Correia-de-Sá, Ricardo Araujo, Fernando Barbosa, Philip W. J. Burnet, Joana Ferreira-Gomes, Benedita Sampaio-Maia

**Affiliations:** ^1^ICBAS – Instituto de Ciências Biomédicas Abel Salazar da Universidade do Porto, Porto, Portugal; ^2^NanoBiomaterials for Targeted Therapies, INEB – Instituto Nacional de Engenharia Biomédica, i3S – Instituto de Investigação e Inovação em Saúde, Universidade do Porto, Porto, Portugal; ^3^Departamento de Biomedicina, Faculdade de Medicina, Universidade do Porto, Porto, Portugal; ^4^Laboratório de Neuropsicofisiologia, Faculdade de Psicologia e de Ciências da Educação, Universidade do Porto, Porto, Portugal; ^5^Department of Psychiatry, University of Oxford, Oxford, United Kingdom; ^6^IBMC – Instituto de Biologia Molecular e Celular, i3S – Instituto de Investigação e Inovação em Saúde, Universidade do Porto, Porto, Portugal; ^7^Faculdade de Medicina Dentária, Universidade do Porto, Porto, Portugal

**Keywords:** gut microbiota, oral microbiota, oral-gut translocation, human microbiome, oral-gut axis, bacterial translocation *Actinomyces*, *Rothia*, *Bacteroides*

## Abstract

**Introduction:**

Although the oral cavity and the gut are anatomically continuous regions of the gastrointestinal tract, research on the relationship between oral and gut microbiota remains sparse. Oral-gut bacterial translocation is mostly studied in pathological contexts, thus evidence of translocation in healthy conditions is still scarce. Studying the oral-gut microbiota relationship in humans in different life stages is necessary in order to understand how these microbial communities might relate throughout life.

**Methods:**

In this study, saliva and fecal samples were collected from healthy participants (39 children, 97 adults). Microbiota analysis was carried out by sequencing the V4 region of the 16S ribosomal RNA gene, followed by amplicon sequence variant (ASV) analysis.

**Results and discussion:**

Although the oral and gut microbiota are vastly different, a subset of 61 ASVs were present in both the oral cavity and gut of the same individual, and represented 1.6% of all ASVs detected. From these, 26 ASVs (classified into 18 genera: *Actinomyces*, *Rothia*, *Bacteroides*, *Porphyromonas*, *Prevotella*, *Alistipes*, *Fusobacterium*, *Neisseria*, *Haemophilus*, *Akkermansia*, *Solobacterium*, *Granulicatella*, *Streptococcus*, *Gemella*, *Mogibacterium*, *Dialister*, *Veillonella*, *Christensenellaceae R-7 group*) were present in both children and adults, suggesting the possibility of persistent colonization of both habitats by these microorganisms, initiating in childhood. Additionally, 62% of shared ASVs were more abundant in the oral cavity, indicating that oral-to-gut translocation may be the main route of translocation between environments, and highlighting that this phenomenon might be more common than previously thought in healthy individuals of all ages.

## 1 Introduction

The human microbiota has become a focus of study in health sciences because of its impact on host physiology and wellbeing. It is composed of bacteria, viruses, archaea, and lower and higher eukaryotes (Hou et al., 2022), although the vast majority of studies focuses on bacteria. A recent estimate suggests a ratio of human:bacterial cells in the body close to 1:1 (Sender et al., 2016; Thursby and Juge, 2017). Microbiota-host interactions are complex and bidirectional and seem to have a significant impact on host health and wellbeing (Shreiner et al., 2015). A healthy microbiota community often demonstrates relative stability and high taxonomic diversity, although the relative distribution of microorganisms is unique between individuals and may undergo variations throughout life (Hou et al., 2022). When the balance of the microbe community and/or the community-host relationship is disrupted, dysbiosis occurs, which can lead to disease (Rinninella et al., 2019).

Microbiota acquisition is shaped by external factors since early life, such as type of delivery, feeding method, weaning period, antibiotic usage, lifestyle, diet, and cultural habits (Thursby and Juge, 2017; Rinninella et al., 2019). Richness and diversity of the early microbiota are crucial for a healthy microbial composition throughout adulthood (Rinninella et al., 2019). Bacterial composition, diversity and even function are affected by age, with the microbiota maturing and stabilizing from childhood into adulthood (Badal et al., 2020; Burcham et al., 2020; Hou et al., 2022; Li et al., 2022). Regarding the maturation of the oral microbiota, a study by Burcham et al. (2020) comparing the oral microbiota of 181 adults and 185 children and adolescents (aged 8–17 years of age) found that oral microbiota composition was more diverse in youth than adults. Pertaining to the gut, a study by Radjabzadeh et al. (2020) comprising 2,111 children (9–12 years of age) and 1,427 adults (46–88 years of age) found that children had significantly lower gut microbiota diversity than adults. A systematic review of 27 empirical human studies further concluded that gut alpha diversity regarding microbial taxa, metabolites, and functional pathways is higher in older adults than younger individuals (Badal et al., 2020). Overall, these studies highlight that the maturation of the human microbiota leads to both compositional and functional differences between children and adults, although data regarding the maturation of oral and other microbiota beyond the gut is still scarce.

The human gastrointestinal tract constitutes one of the largest interfaces between the host and external factors and is thought to encompass more than 1,014 microorganisms and 50–100 times the amount of genomic content as the human genome (Bäckhed et al., 2005; Gill et al., 2006; Thursby and Juge, 2017; Kho and Lal, 2018). The gut microbiota provides several local beneficial properties to the host, such as regulating digestion, playing a role in nutrient extraction, synthesis, and absorption (including vitamins, lipids, amino acids, and short-chain fatty acids), maintaining the integrity of the intestinal epithelium, protecting against pathogens by producing bacteriocins or competing for resources, and playing an essential role in host immune function (Thursby and Juge, 2017; Rinninella et al., 2019). Considering this significant impact on host physiology, it is not surprising that gut dysbiosis is associated with several diseased states, with findings pointing to a role in hypertension, cardiovascular diseases, inflammatory bowel diseases, obesity, some types of cancer and, most recently, neurodegenerative conditions (such as Alzheimer’s and Parkinson’s disease) and mental health disorders (Afzaal et al., 2022; Ullah et al., 2023). The oral cavity is home to the second largest and most diverse microbial community after the gut. In addition to being the initial point of digestion, the oral microbiota is essential in maintaining both oral and systemic health (Deo and Deshmukh, 2019). Oral dysbiosis induces oral infectious diseases, such as caries, periodontal disease, and oral candidiasis, and seems to play a significant role in the development of certain types of cancer (oral, pancreatic, genitourinary, and gastrointestinal), hypertension, rheumatoid arthritis, systemic lupus erythematosus, and even Alzheimer’s disease and mental health disorders (Xie et al., 2021; Hou et al., 2022; Baker et al., 2024).

As anatomically continuous regions encompassed in the gastrointestinal tract, the oral cavity and the gut are physically and chemically linked (Park et al., 2021). Despite this, research on the oral and gut microbiota is still mostly conducted in an organ-specific manner, rather than in an integrative way (Park et al., 2021). Evidence shows that oral bacteria can translocate into the gut, especially in individuals where chemical hurdles (bile and gastric acid) are weakened, like in infants or the elderly (Park et al., 2021). For example, in newborns, gut-resident *Bifidobacterium*, the most abundant bacterial genus in the neonatal gut, has also been detected in their oral fluid (Toda et al., 2019; Park et al., 2021). Similarly, in elderly people, the prevalence of oral bacteria in the gut is higher than in younger adults (Odamaki et al., 2016; Iwauchi et al., 2019; Park et al., 2021). Transmission of bacteria can also occur through the fecal-oral route in particular circumstances, such as unsanitary settings or immunocompromised conditions (Gaetti-Jardim et al., 2018; Shaffer and Lozupone, 2018; Ayele et al., 2019; Park et al., 2021). Bacterial translocation between these habitats seems to have an impact on host health: a recent study by Kunath et al. (2022) reported that gut microbiota changes driven by alterations in mouth-to-gut bacterial transfer may contribute toward the inflammatory processes involved in type 1 diabetes mellitus, further highlighting the urgent need to understand how the oral-gut microbiota relate to each other and their joined impact on host health. Even though evidence suggests that the oral cavity and the gut are closely connected through oral-to-gut and fecal-to-oral routes (Park et al., 2021), these microbial communities are rarely studied simultaneously in the same individual and evidence on their relation outside of pathological contexts is still scarce.

Taking all that was mentioned into consideration, this study aimed to (a) characterize the oral and gut microbiota of a cohort of healthy children and healthy adults as a way to explore the oral-gut microbiota relationship and the possibility of bacterial translocation in healthy conditions, and (b) explore how this relationship might vary with age.

## 2 Materials and methods

### 2.1 Participants

Healthy children (*n* = 39) and healthy adults (*n* = 97) were selected from cohorts from ongoing studies (M2Child and Microbi-A cohorts) based on the inclusion and exclusion criteria listed below. All participants were recruited from the community through press releases, university mailing lists, word of mouth, and web advertisements. Children were evaluated in Faculdade de Medicina da Universidade do Porto (Porto, Portugal), while adults were evaluated in Instituto de Investigação e Inovação em Saúde da Universidade do Porto (i3S, Porto, Portugal). Relevant clinical and demographic information (age, sex, weight, height, probiotic intake, clinical history, medication intake, oral health history and habits, smoking habits, and education level – these last three factors collected only for adults) was gathered for each participant through a semi-structured interview. Exclusion criteria for both groups included inability to give/obtain informed consent or lack of parental/guardian consent (in the case of children), recent history of antibiotic therapy (less than 3 months for adults and 6 months for children) and recent history of probiotic intake (<3 months). For adults, age over 65 years old, smoking, pregnancy, obesity (Body mass index – BMI ≥ 30.0), and active oral health conditions at the time of sampling (caries, periodontitis, candidiasis, canker sores, and other infections) also constituted exclusion criteria.

The study protocols for M2Child and Microbi-A were approved by the Ethics Committee for Health of Centro Hospitalar Universitário de São João (approval number 318/2020) and the Ethics Committee for Health of Faculdade de Psicologia e de Ciências da Educação da Universidade do Porto (approval number 2020/12-01b), respectively, and followed the 1964 Helsinki declaration and its later amendments; all participants were volunteers and written informed consent was obtained from the participant or the legal guardian (in the case of children).

### 2.2 Sample collection

Fecal samples were collected by the participants/legal guardians at home into 60 mL sterile fecal collection containers previously provided by the research team. The participants were instructed on how to properly collect the sample without urine contamination and to keep the sample frozen until delivery to the lab. The samples were delivered frozen and on ice in thermal bags previously provided. Sample collection took place as close as possible to the day of evaluation at Universidade do Porto (mostly the day before or on the day of evaluation).

Unstimulated whole saliva was collected on the day of evaluation by the members of the research team via the spitting method (Navazesh, 1993) into sterile containers. Prior to the collection, participants did a water mouthwash in order to clean the oral cavity. Saliva collection began after swallowing the residual saliva present in the mouth and allowing newly produced saliva to accumulate.

Saliva and fecal samples were collected from the same participant no more than 3 days apart. Both saliva and fecal samples were immediately stored at −80°C.

### 2.3 DNA extraction and 16S rRNA gene sequencing

DNA was isolated from stool and saliva samples after optimisation of the extraction process. DNA extraction from stool samples was done utilizing the DNeasy Blood & Tissue Kit (Qiagen, Germany), following a modified version of the protocol of the QIAamp DNA Stool Mini Kit (Qiagen, Germany) described in the QIAamp DNA Stool Handbook (Qiagen, 2010) available on the Qiagen website. All reagents were used as provided in the DNeasy Blood & Tissue Kit. InhibitEX Buffer (Qiagen, Germany) and ethanol for molecular biology (Merck, Germany) were purchased separately. The step-by-step modified protocol is described in the Supplementary material.

DNA extraction from saliva samples was also done utilizing the DNeasy Blood & Tissue Kit, following a modified version of the “User-Developed Protocol: Purification of total DNA from animal saliva using the DNeasy^®^ Blood & Tissue Kit” (Qiagen, 2006) available on the Qiagen website. All reagents were used as provided in the DNeasy Blood & Tissue Kit. Ethanol for molecular biology was purchased separately. The step-by-step modified protocol is described in the Supplementary material.

For each sample, 3 independent amplifications of the V4 region of the 16S rRNA gene were performed in 384 well plates and were then combined in a final pool (Earth Microbiome Project, 2018), using the 515F/806R (V4 region updated sequences: 515F (Parada)–806R (Apprill), forward-barcoded: FWD:GTGYCAGCMGCCGCGGTAA; REV:GGACTACNVGGGTWTCTAAT) primer pairs recommended by the Earth Microbiome Project (2018). An index sequence was incorporated into the forward primer in order to distinguish between samples of a single PCR reaction. Amplification was carried out under the following PCR cycling conditions: 94°C for 3 min; 35 cycles of 94°C for 60 s, 50°C for 60 s and 72°C for 105 s; and extension at 72°C for 10 min. After PCR pooling and library preparation, sequencing was performed at the Instituto Gulbenkian de Ciência (IGC) Genomics Unit using an Illumina MiSeq Sequencer and MiSeq Reagent Kit v3 producing 2×250 bp reads. Sample demultiplex was performed using QIIME 2 demux script.

### 2.4 Data and statistical analysis

Amplicon sequence variants (ASVs) were identified using a previously suggested R pipeline and DADA2 method (Callahan et al., 2016). Primer v7 (PRIMER-e, Auckland, New Zealand) was used for the calculation of diversity indices, namely the Shannon index, as well as non-metric multidimensional scaling (NMDS), principal coordinate analyses (PCO), and other multivariate analyses used to test the significance of *β*-diversity, such as analysis of similarities (ANOSIM). The number of reads in each sample was initially converted into percentage values to eliminate the effect of the variability of the final number of reads among samples (Araujo et al., 2019). The percentage of each amplicon sequence variant (ASV) per sample was used for these analyses, followed by square-root transformed data, resemblance matrices of similarity data types using Bray-Curtis similarities, adding dummy value, and testing 4,999 permutations. Post-hoc analyses were done in STAMP 2.1.3 (Parks and Beiko, 2010) and the statistical tests used for two groups were analysed using Welch’s *t*-test (two-sided, Welch’s inverted for confidence interval method).

## 3 Results

### 3.1 Participant characterization

Demographic information relating to both children and adult participants is included in Table 1. Children’s ages ranged from 5 to 10 years old, with an average age of 7.7 ± 1.6 years, while adults ranged from 20 to 61 years old, with an average age of 38.1 ± 10.9 years. BMI information was recorded for 31 children (out of 39; parents were unable to provide updated information for 8 children). Children had a mean BMI of 16.8 ± 2.8 kg/m2 and a mean z-score BMI-for-age of 0.36 ± 1.31: 0% underweight, 77% normal weight, 10% overweight, and 13% obese, according to the WHO BMI-for-age charts for girls and boys between 5 and 19 years (World Health Organization, 2007). Adults had a mean BMI of 23.2 ± 2.7 kg/m2: 4% underweight, 68% normal weight, 28% overweight, and 0% obese, according to the WHO BMI classification (World Health Organization, 2010). Ninety per cent of adult participants pursued higher education (at university level), while 10% received education up to grade 12.

**Table 1:** Participant demographics.

	Children (*n* = 39)	Adults (*n* = 97)
Age (years)		
Median (IQR)	8.0 (7–9)	40.0 (28–47)
Sex (male %)	56.4%	32.0%

IQR, interquartile range.

### 3.2 Gut vs. oral microbiota

Of the large set of ASVs described in gut and oral samples, 2,424 were found in gut samples, while 1,438 ASVs were reported in oral samples. A total of 2,329 ASVs were detected exclusively in the gut (absent from the oral cavity), while 1,343 ASVs were exclusively detected in the oral cavity (absent from the gut). Interestingly, there was a subset of 95 ASVs commonly found in gut and oral samples of multiple individuals. Among these, a total of 61 ASVs were found simultaneously in both the gut and oral samples collected from the same individual. At least one of these shared ASVs (present in the oral cavity and gut of the same individual) was detected in 96% of the total population (131 individuals), meaning only five individuals (out of 136) did not exhibit shared ASVs between the oral cavity and the gut.

**Figure 1:**
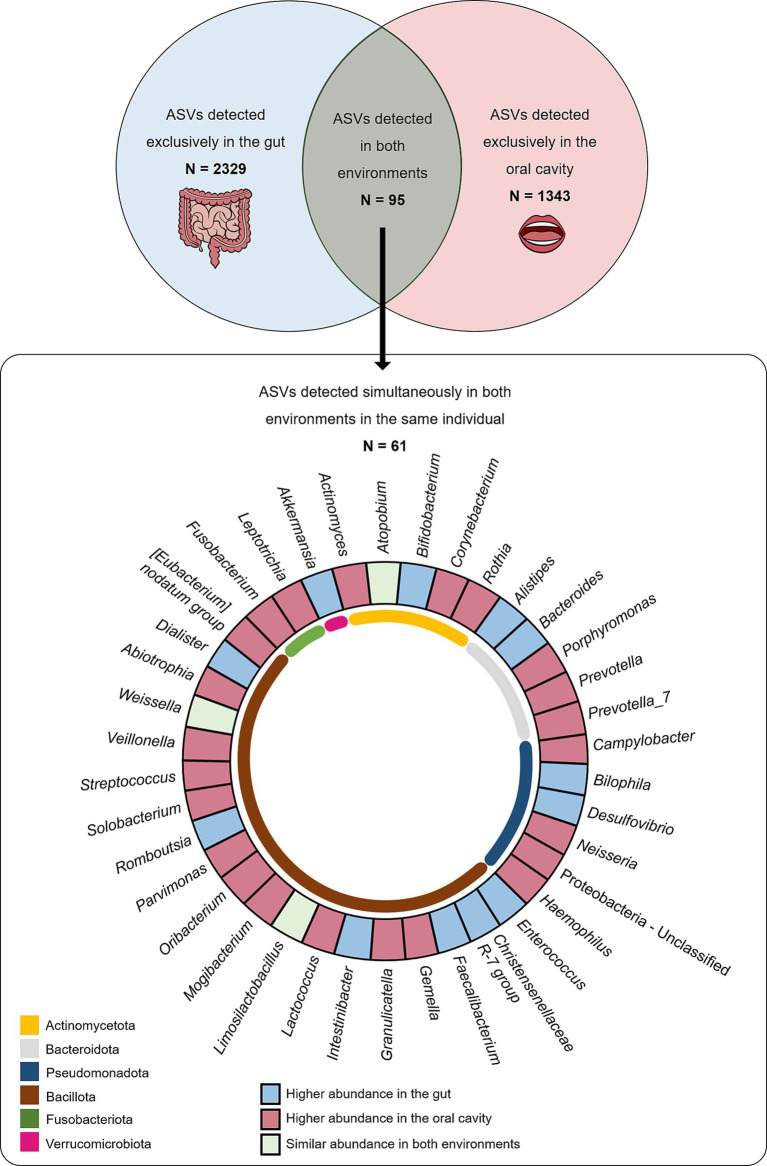
The number of ASVs detected exclusively in gut (light blue) and oral (light pink) samples and number of ASVs detected in both samples (light green) are displayed in the top panel. The number of ASVs detected in both samples from the same individual are displayed in the bottom panel according to taxa and where they are most abundant.

By analysing the frequency values of these 61 ASVs found simultaneously in both the gut and oral habitats, it was possible to observe that 38 ASVs (62%), classified into 23 genera, were more abundant in oral samples than in gut samples, while only 17 ASVs (28%), classified into 12 genera, were more abundant in gut samples than in oral samples (Figure 1). No significant differences could be observed among this subset of shared ASVs regarding sex or BMI categories.

### 3.3 Children vs. adults microbiota

**Figure 2:**
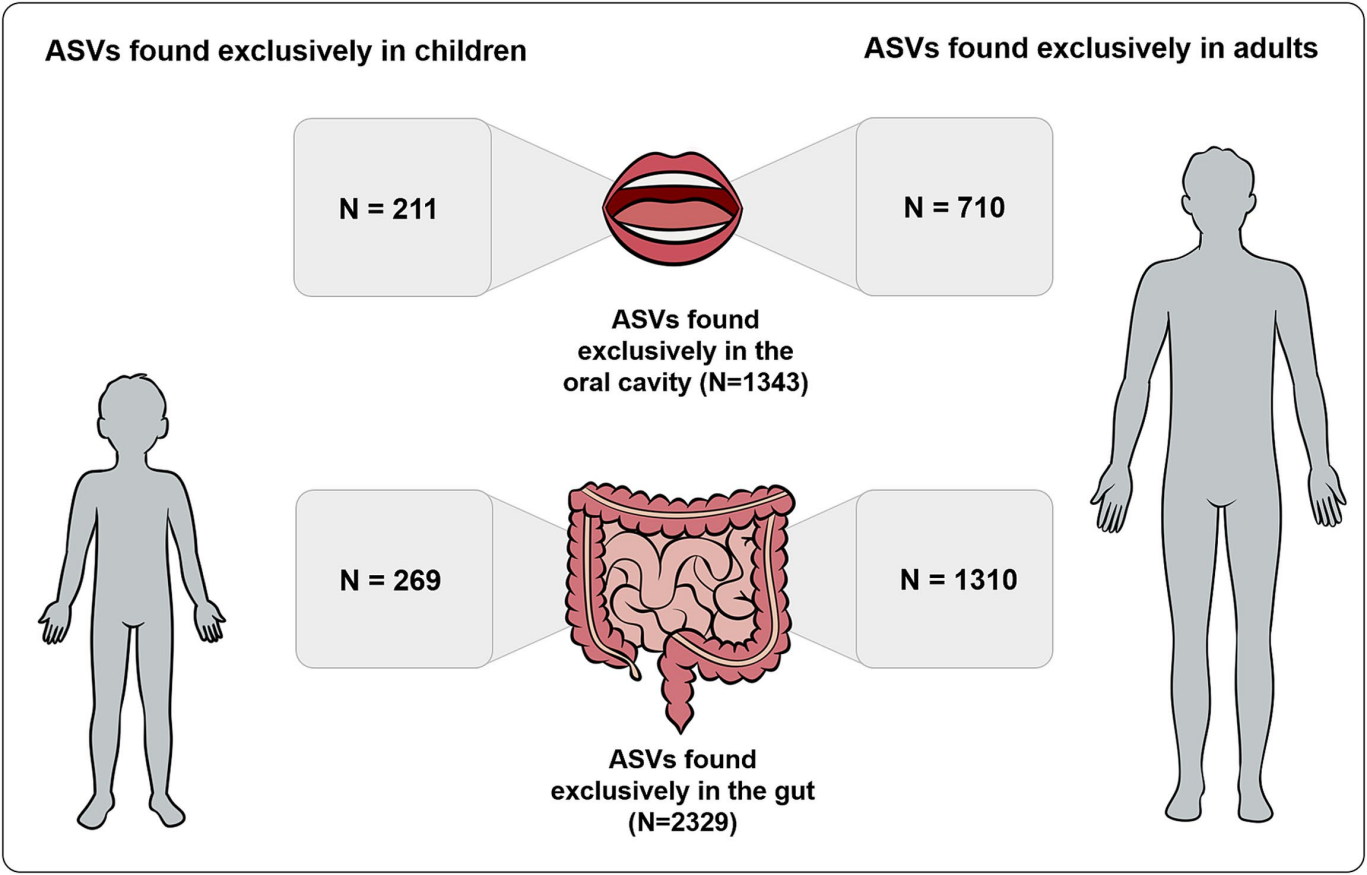
Number of ASVs detected exclusively in gut and oral samples also found exclusively in children and adults.

A total of 480 ASVs were detected exclusively in children, while a total of 2020 ASVs were detected exclusively in adults. From the 2,329 ASVs detected exclusively in the gut, 1,310 ASVs were present exclusively in adults and 269 exclusively in children. From the 1,343 ASVs detected exclusively in the oral cavity, 710 ASVs were present exclusively in adults and 211 ASVs exclusively in children (Figure 2).

**Figure 3:**
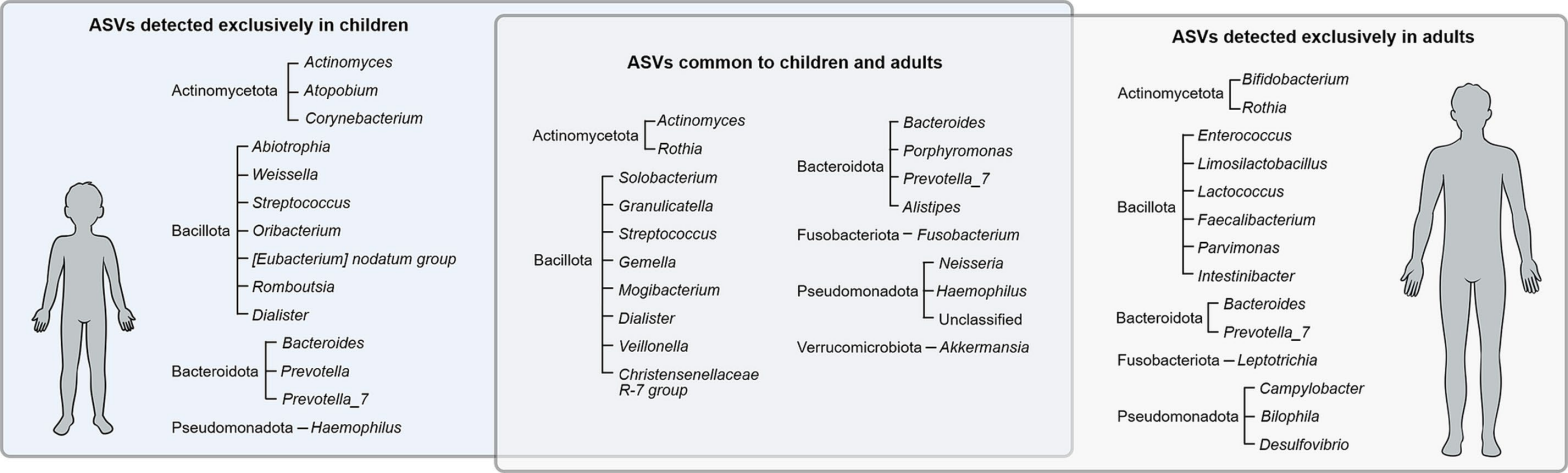
Bacterial ASVs (phylum and genera) detected exclusively in children, exclusively in adults, and common to both groups.

From the 61 ASVs found simultaneously in both the gut and oral habitats, 20 ASVs were exclusively found in adults (classified as Actinomycetota, Bacteroidota, Pseudomonadota, Bacillota, and Fusobacteriota), while 15 ASVs were exclusively found in children (classified as Actinomycetota, Bacteroidota, Bacillota, and Pseudomonadota) (Figure 3). The remaining subset of 26 ASVs were found in samples from both adults and children and were classified as Actinomycetota, Bacteroidota, Bacillota, Fusobacteriota, Pseudomonadota and Verrucomicrobiota.

### 3.4 Bacterial diversity

**Figure 4:**
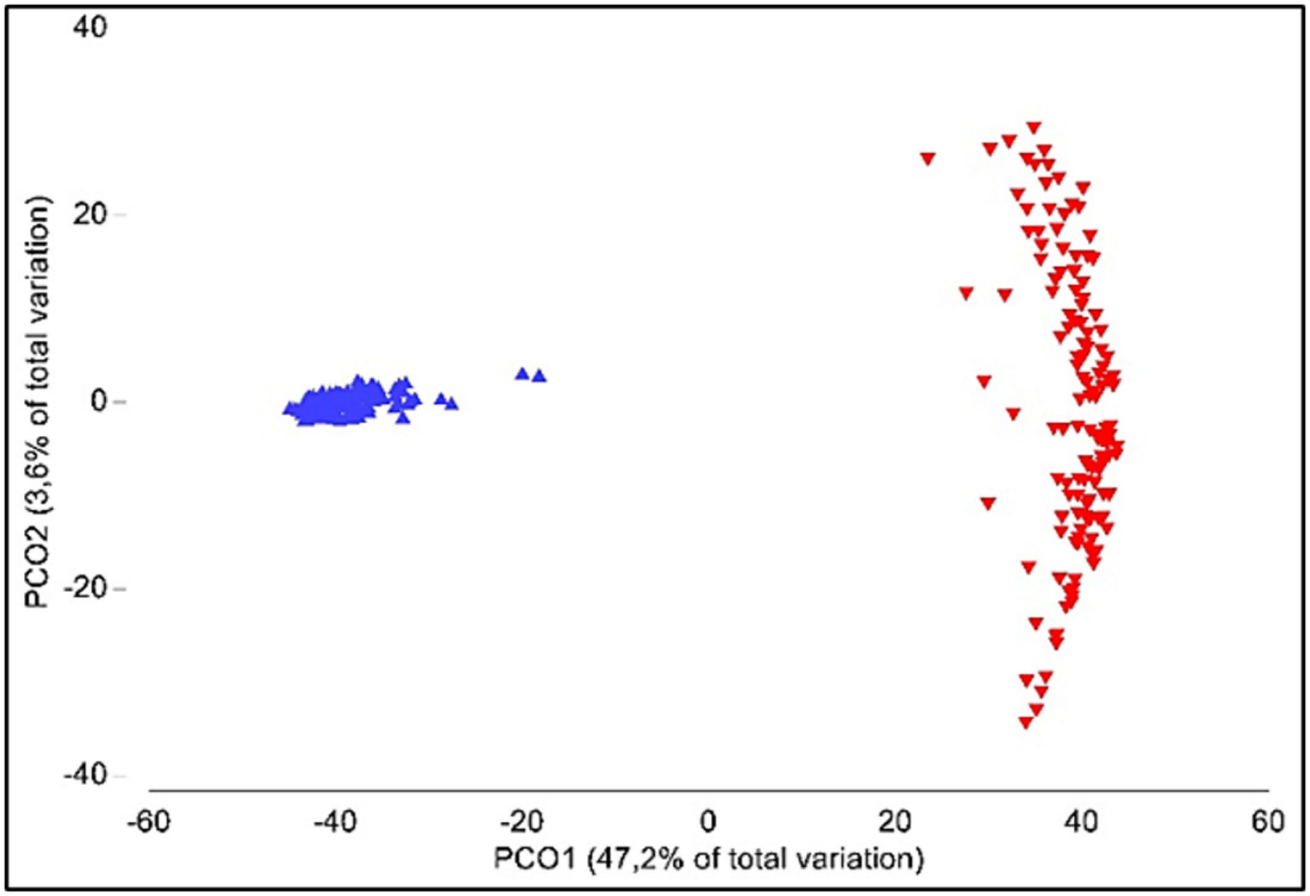
Principal coordinate analyses (PCO) of gut (blue) and oral (red) microbiota.

Alpha-diversity was significantly different between oral and gut samples (*p* < 0.001); average Shannon index values were 4.77 ± 0.42 (ranging from 3.98 to 5.45) and 4.45 ± 0.37 (ranging from 3.68 to 5.38) for gut and oral samples, respectively. Analysis of similarities (ANOSIM) showed significant differences when comparing the gut and oral microbiota (*β*-diversity; *p* < 0.001) (Figure 4).

**Figure 5:**
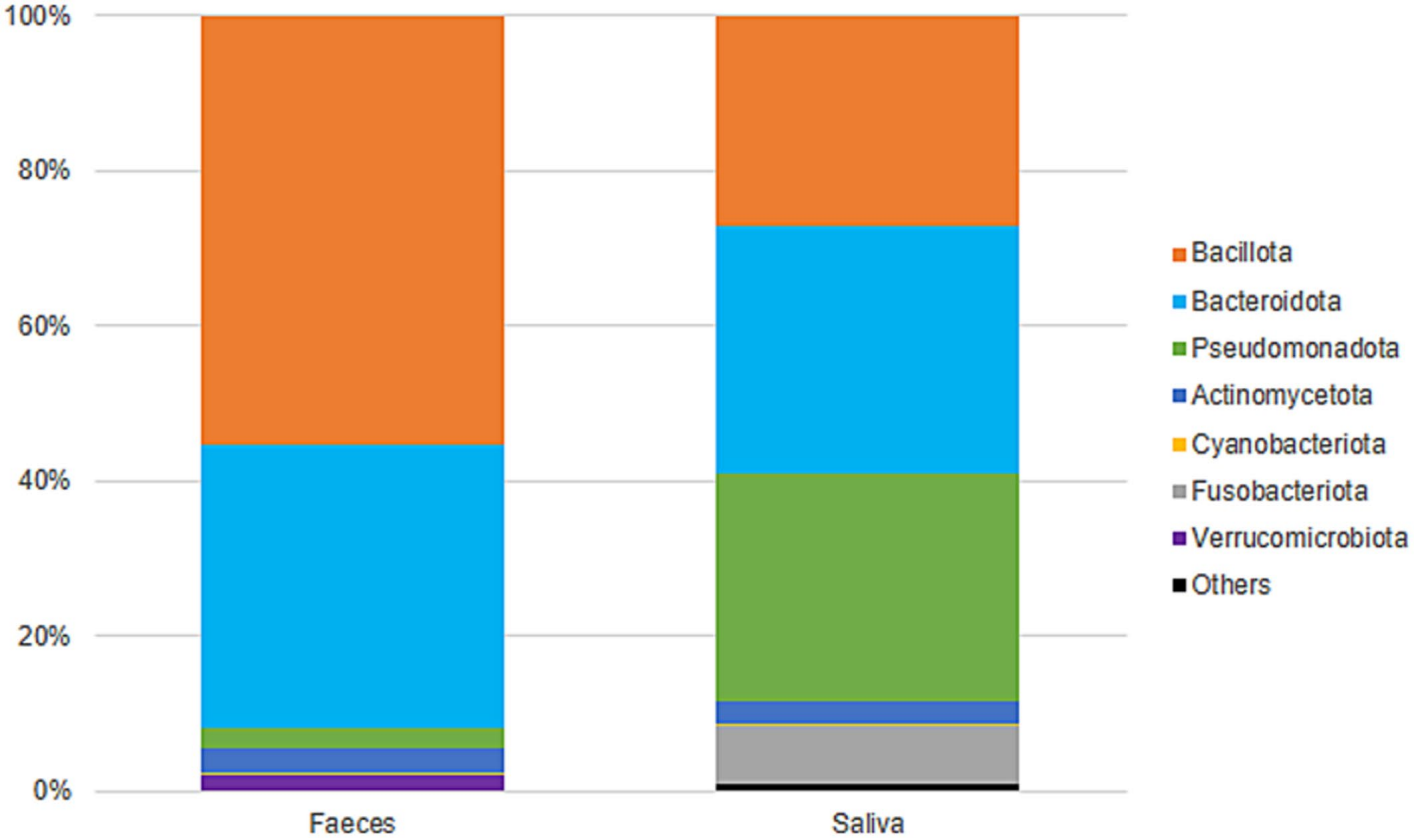
Taxonomic classification (phyla) and distribution of gut and oral samples.

Gut samples were dominated by Bacillota and Bacteroidota, while oral samples were dominated by Bacteroidota, Pseudomonadota, and Bacillota (Figure 5). At the family level, gut samples included Lachnospiraceae (23%), Bacteroidaceae (19%), Ruminococcaceae (13%), Prevotellaceae (8%), and Rikenellaceae (5%), among others. Oral samples were predominantly colonized by Prevotellaceae (27%), Pasteurellaceae (15%), Neisseriaceae (13%), Veillonellaceae (11%), and Streptococcaceae (10%) (Supplementary Figure 1). Gut diversity and oral diversity values do not seem to be correlated (R2 = 0.0303) in our study population (Supplementary Figure 2).

#### 3.4.1 Bacterial diversity with regards to age

**Figure 6:**
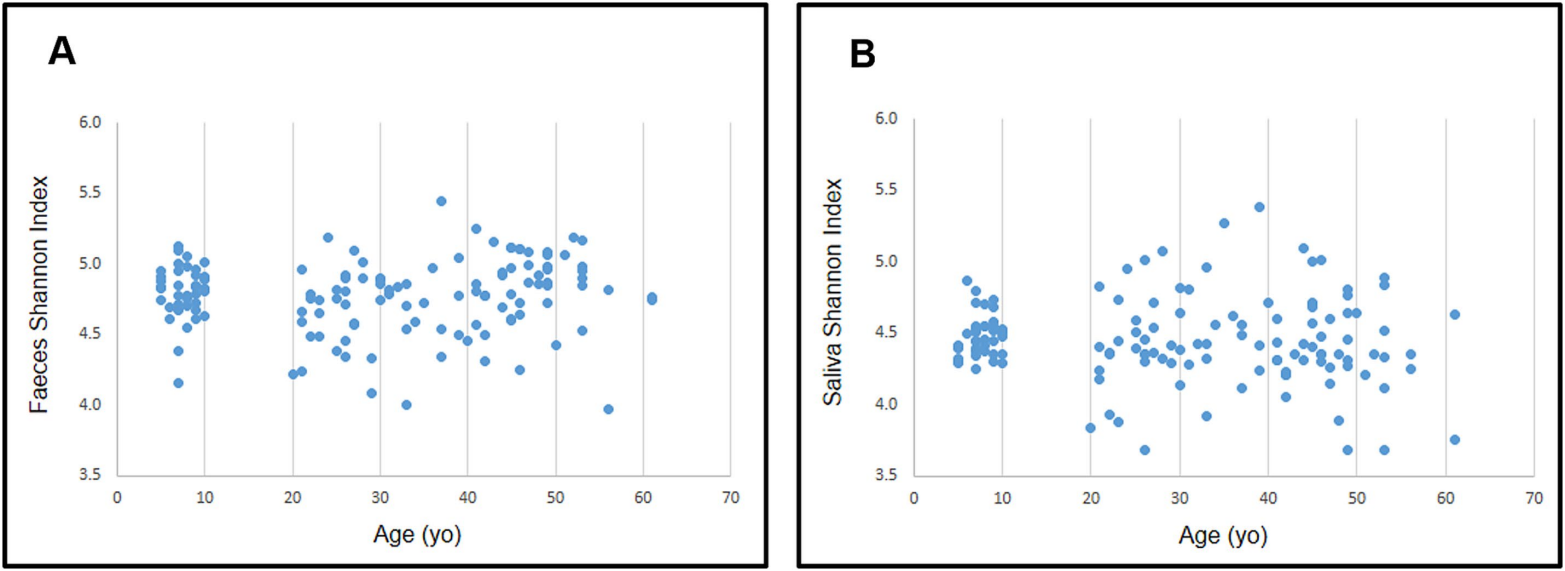
Distribution of Shannon diversity index for gut (A) and oral (B) microbiota according to participant’s age.

**Figure 7:**
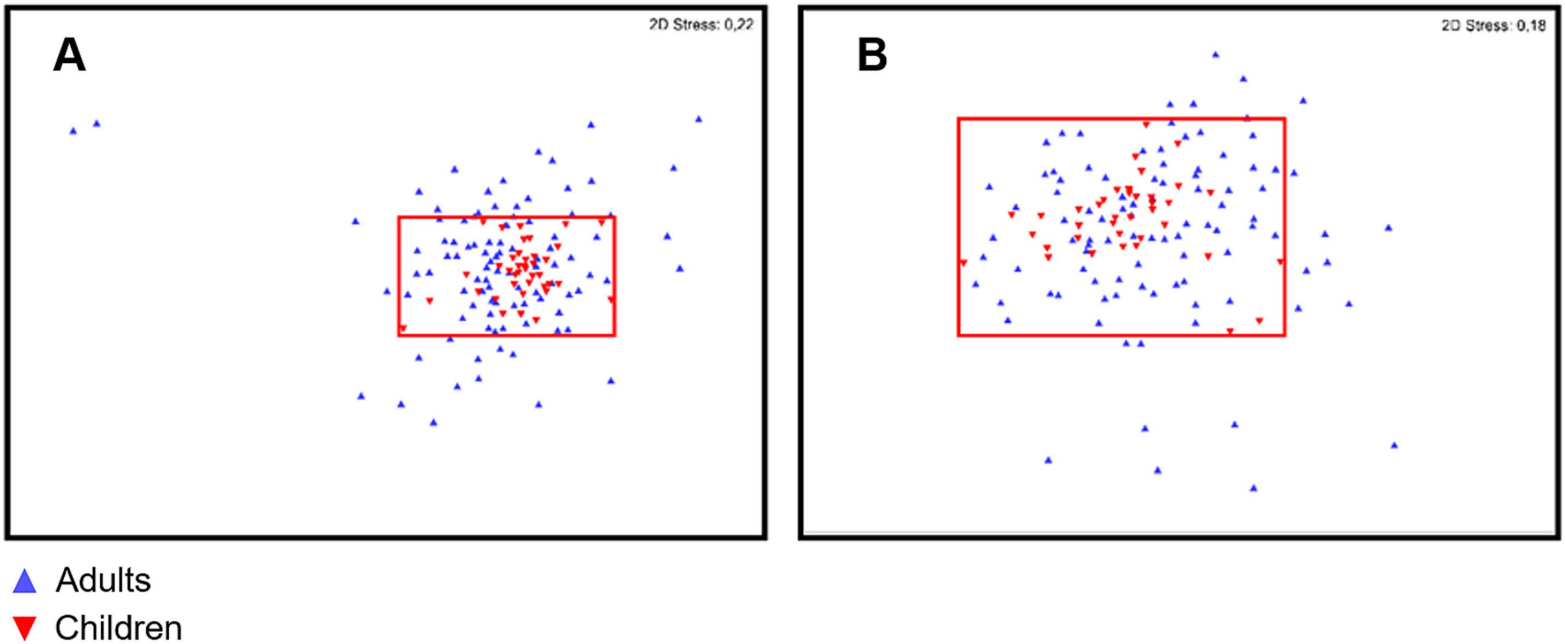
Non-metric multidimensional scaling (nMDS) of gut (A) and oral (B) microbiota for both children (red) and adult (blue) populations. Red area marks the distribution and limits of diversity observed for children gut and oral microbiota.

No differences were found in either gut or oral samples when comparing adult vs. child microbiota for both *α*- and *β*-diversities (*p* = 0.468 and 0.482 respectively; Figure 6 for α-diversity; Figure 7 for β-diversity). Details on taxa can be found in Supplementary Figure 1. Nevertheless, it was possible to visualize a more uniform distribution of gut and oral samples from children, while the adults’ samples were more dispersed within each group (Figures 6, 7).

Considering multiple age groups, gut and oral microbiota did not differ in relation to α-diversity, but differences were observed for β-diversity of children microbiota (5–10 years old) vs. older adults (over 45 years old; *p* < 0.050 for these comparisons in both environments). Details on taxa can be found in Supplementary Figures 3, 4. When comparing age groups in adulthood (≤ 44 years old vs. ≥ 45 years old), although no differences were observed for β-diversity, there was a significant difference in the α-diversity of adults’ gut microbiota (*p* = 0.022).

#### 3.4.2 Bacterial diversity with regards to sex

**Figure 8:**
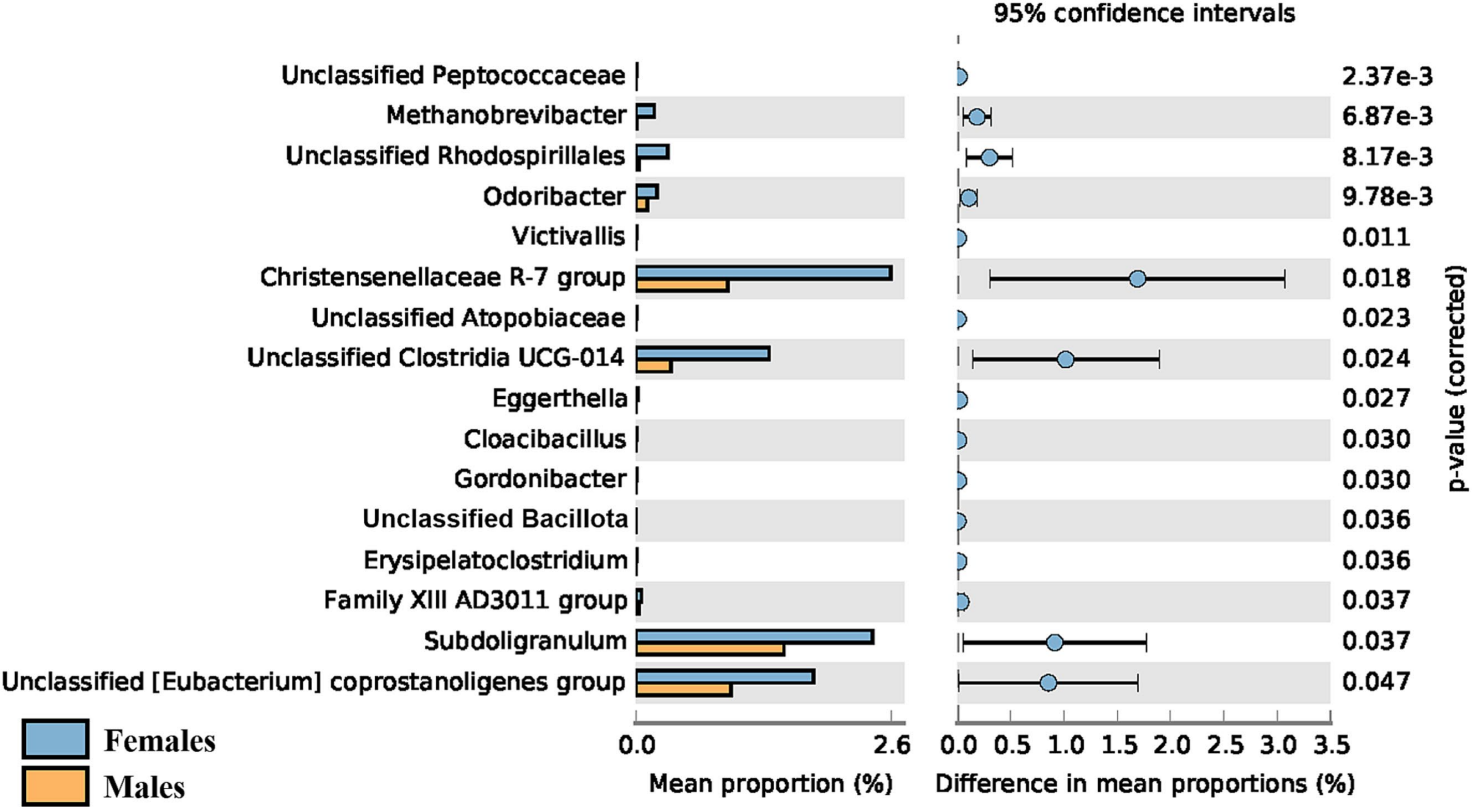
Post-hoc analyses showing differences on taxonomic groups of gut microbiota between male and female young adults (<45 years old).

Regarding sex, a significant difference in the α-diversity of the gut microbiota was observed when comparing young adult (< 45 years old) female and male participants (*p* = 0.016), with the average Shannon index being higher in females (4.81 ± 0.26 vs. 4.66 ± 0.29). However, this difference was only observed in younger adults and was no longer detectable in adults over 45 years old. No sex-driven differences were observed in the gut or oral microbiota of children (*p* = 0.959 and 0.882 respectively), nor in the oral microbiota of adults. The taxonomic differences between female and male young adults (< 45 years old) can be seen in Figure 8, with female participants showing greater abundance of all 16 genera identified.

#### 3.4.3 Bacterial diversity with regards to BMI

**Figure 9:**
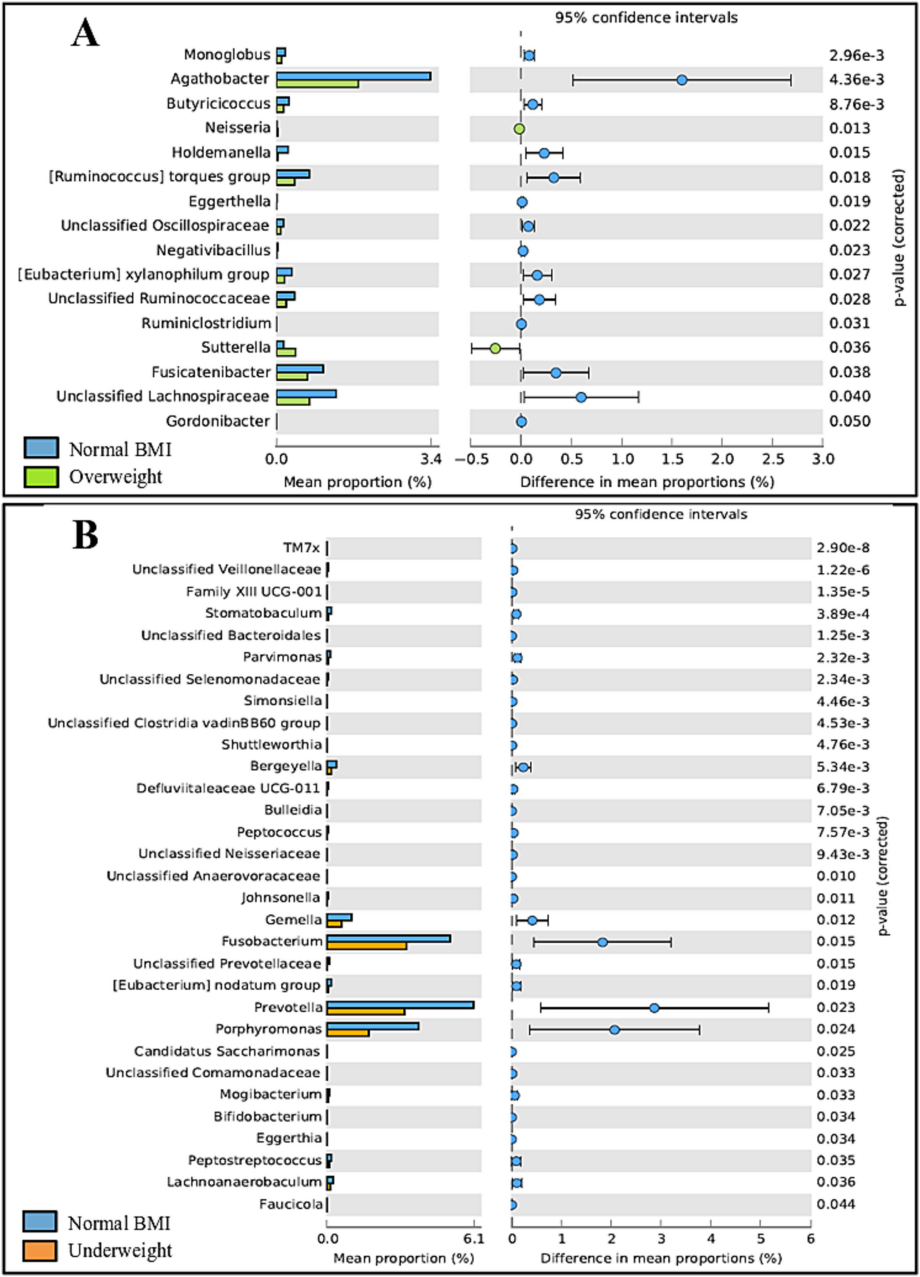
Post-hoc analyses showing differences on taxonomic groups of gut (A) and oral (B) microbiota across BMI groups.

Some differences were observed regarding BMI in adults. Gut microbiota differed significantly between adults classified as overweight vs. normal BMI (*p* = 0.040), with the normal weight group exhibiting a higher abundance of 14 out of 16 genera. Oral microbiota showed significant differences between adults classified as underweight vs. normal BMI (*p* = 0.034), with the underweight group exhibiting a lower prevalence of all 31 genera identified; details on taxonomic differences can be seen in Figure 9. Of note, no obese adults were included in the study.

No differences regarding BMI were detected in children, neither for the gut nor oral microbiota.

#### 3.4.4 Other variables

No significant differences (all *p* > 0.050) were observed regarding the adults’ educational level (up to grade 12 vs. higher education) nor based on oral hygiene habits (tooth brushing frequency).

## 4 Discussion

This work explored the oral-gut microbiota relationship in healthy children and adults. We have found a residual (0.7%) but persistent (in children and adults) set of shared taxa between oral and gut habitats. Most of the study participants (>95%) presented at least one shared oral-gut taxa, showcasing that, although gut and oral microbiota differ vastly, specific bacterial taxa may be commonly shared between oral and intestinal habitats in humans.

The fact that the vast majority of the ASVs detected in this study is exclusive to each environment reveals an unsurprising high habitat specificity of microbiota. Indeed, 96% of ASVs originating in gut samples were exclusive to this habitat (absent from the oral cavity), while 93% of ASVs originating in the oral milieu were exclusively found in this body-site (absent from the gut).

Regarding different age groups, 12% of ASVs were detected exclusively in children, while 52% of ASVs were detected exclusively in adults, highlighting differences in composition between life stages. This suggests an age-related maturation of the oral and gut microbiota, which seems to encompass both the loss of certain microorganisms and the acquisition of new ones. However, it should be noted that our study only included 39 children and a larger sample size would allow for clearer conclusions. Despite the differences, a subset of shared ASVs was identified: from the 61 ASVs common to the gut and oral cavity of the same individual, 43% of these were also shared between children and adults, classified as 6 phyla (Actinomycetota, Bacteriodota, Fusobacteriota, Pseudomonadota, Verrucomicrobiota, and Bacillota). It is possible that the proportion in which these ASVs (common in childhood and adulthood) are shared between habitats might actually fluctuate between life stages due to both external and internal factors (such as the influence of hormones after puberty) instead of remaining stable throughout life. A longitudinal study would allow the exploration of this hypothesis. Interestingly, the number of shared oral-gut ASVs was higher in adult subjects, which is in agreement with previous observations (Kageyama et al., 2023) reporting an increased abundance of shared ASVs with age. According to the authors, this may be due to a heightened translocation of oral bacteria to the gut with age (Kageyama et al., 2023).

The presence of common bacteria in both the oral cavity and the gut has been previously reported in other studies as well (Segata et al., 2012; Schmidt et al., 2019) and strongly suggests translocation between the two body-sites. Additionally, the fact that the majority of ASVs (62%) is more abundant in the oral cavity suggests that oral-to-gut transmission might be the main route of translocation between these two habitats and that, despite being mostly explored in a pathological context (Strauss et al., 2011; Huh and Roh, 2020; Lu et al., 2023; Liao et al., 2024), this phenomenon might be more frequent than previously thought in healthy individuals (Schmidt et al., 2019; Park et al., 2021).

Even though significant amounts of oral bacteria are constantly swallowed throughout the day along with saliva and bolus (Humphrey and Williamson, 2001), the oral cavity and the gut are separated by several barriers (gastric acidity, bile acid, the host immune system, competition with native bacteria in the gut) that, in healthy conditions, cause the majority of oral bacteria to be inactivated (Park et al., 2021; Kageyama et al., 2023; Lu et al., 2023). For this reason, it is not surprising that most microorganisms that “depart” from the oral cavity are unable to reach the gut in a viable form. Also, those which do reach the gut in a viable form must adapt to the conditions in the gut efficiently enough to colonize this habitat. These factors would explain why only 1.6% of all ASVs detected in our study are shared between the oral cavity and the gut of the same individual, and only 0.7% are shared between environments both in childhood and adulthood.

In our sample, only five participants (all adults, two females and three males) exhibited no shared ASVs between habitats. None of these participants reported any relevant clinical history (with only one reporting an allergy to dust), none took antibiotics in the 6 months prior to sample collection, none reported food restrictions (vegetarian diet, etc.) and none have ever taken probiotics. Therefore, the absence of shared ASVs does not seem to be explained by nutritional reasons or past disease history. Further studies would be necessary in order to understand why no shared ASVs were detected in these individuals. Perhaps including several timepoints would clarify if this absence of shared ASVs is a constant feature in these participants or if it is a transitory condition. One could also hypothesize that these adults could have a lower pH of stomach acid (either permanently or at the time of sampling), which would be more effective in reducing the viability of oral bacteria and avoid oral-gut translocation, but medical studies should be performed in order to confirm this theory. It is also possible that a more extensive sequencing analysis (increased sequencing depth) with more reads obtained per sample would allow for shared ASVs to be detected in these individuals.

In regards to bacterial diversity, greater diversity was observed in gut samples in comparison to oral samples, which is in agreement with previous works reporting that the gut microbiota is more diverse than the oral microbiota (Deo and Deshmukh, 2019; Hou et al., 2022). Gut samples were dominated by Bacillota and Bacteroidota, and oral samples were dominated by the phyla Bacteroidota, Pseudomonadota, and Bacillota, in accordance to what is reported in the literature (Deo and Deshmukh, 2019; Rinninella et al., 2019; Hou et al., 2022). However, in our study, the phylum Bacillota (previously Firmicutes) seems to be somewhat underrepresented in oral samples. The oral cavity is reportedly dominated by Bacillota (Burcham et al., 2020; Park et al., 2021), but in our study the phyla Bacteriodota and Pseudomonadota seem to be slightly more abundant. This could be due to bias in the DNA extraction method utilized, as some methods might exhibit poor ability to capture Gram-positive DNA, leading to an overrepresentation of Gram-negative bacteria or to an underrepresentation of Gram-positive bacteria and consequently affecting the profiling of bacterial communities.

To understand how age would affect diversity, we compared gut and oral diversity of children and adults. Although some studies in the literature report significant differences in diversity between the microbiota of children and adults (Burcham et al., 2020; Radjabzadeh et al., 2020; Ruiz-Ruiz et al., 2020), in this study no significant differences were found in regards to *α*- and *β*-diversities in either the gut or oral microbiota of children compared to adults as a whole. However, we observed that children exhibited a more uniform distribution of gut and oral samples than adults, which may indicate that children have a more similar microbiota to each other while adults might have higher intra-group (person-to-person) variation. These findings are in agreement with a study by Burcham et al. (2020) reporting adult communities to have more intra-group variation in the oral microbiota than youth communities (8–18 years of age). It should, however, be noted that the absence of differences between age groups could also be due to the lack of special specificity, as our bacterial taxonomic characterization was only possible down to the genera level. Thus, although no significant differences are reported at the genera level, a more specific bacterial characterization to the species and strain levels should be explored.

The fact that no differences were observed when comparing our group of children (older than 5 years of age) and adults as a whole may be justified on the grounds that, around 3 years of age, the human microbiota gradually matures, stabilizes and becomes similar to the “adult-like” microbiota (Rinninella et al., 2019; Wu et al., 2021; Brooks et al., 2023). However, when looking at multiple age groups, gut and oral microbiota were different for children and older adults (age group over 45 years of age). It is reported that, although generally stable during adulthood, the human microbiota suffers changes in diversity in older age, possibly due to a decline in immune function and age-related deterioration of intestinal barrier function (in the case of the gut microbiota) (Kim and Benayoun, 2020; Brooks et al., 2023). In fact, changes in microbiota diversity have been reported from 50 to 60 years of age onwards, both regarding the oral microbiota and the gut (Kim and Benayoun, 2020; Willis et al., 2022; Kazarina et al., 2023). It is important to note, however, that both study cohorts (M2Child and Microbi-A) had restrictions regarding age inclusion: only children between 5 to 10 and adults between 18 and 65 years old were included in the original cohorts. This study did not include any adults over the age of 61, thus not allowing us to explore how the oral and gut microbiota may vary between old age (≥ 65 years old) and the age groups already included in this study.

A significant difference in the gut *α*-diversity of adults ≤44 years of age vs. ≥ 45 years of age was detected. Given the higher ratio of female to male participants in our adult population, we decided to test how sex affected the diversity of the microbiota. No sex-driven differences were detected in the gut or oral microbiota of children, nor in the oral microbiota of adults. However, gut α-diversity was significantly higher in female compared to male participants, but only in younger adults, as this difference is no longer detected in adults over the age of 45. Taxonomic differences were also observed in this age group between female and male participants, with the former showing greater abundance of all 16 genera identified. It has been reported that significant sex-driven differences in gut diversity are detected after puberty, but not in non-pubertal subjects (Yuan et al., 2020; Valeri and Endres, 2021), which might explain why we found no significant differences in children. Regarding adults, a study by de la Cuesta-Zuluaga et al. (2019) reported that young adult females (20–45 years of age) from different geographical regions (United States, United Kingdom, and Colombia) exhibited higher α-diversity in comparison to men of the same age, which is in agreement with our results. Sex-associated differences in gut α-diversity seem to be more pronounced in younger adults than in middle-aged adults, with no differences observed in older adults (Haro et al., 2016; Valeri and Endres, 2021). This could be explained by the decline in estrogens levels shown by women in the menopausal phase (Scavello et al., 2019), generally occurring between 45 and 55 years for women worldwide (World Health Organization, 2022). In fact, a recent review on the effect of menopause or female sex hormones on the gut microbiota (Peters et al., 2022b) found decreased α-diversity in post- vs. pre-menopausal women (Flores et al., 2012; Zhao et al., 2019; Peters et al., 2022a) and higher similarity to the male gut microbiota for post-menopausal women (Santos-Marcos et al., 2018; Mayneris-Perxachs et al., 2020; Peters et al., 2022a), which might also explain why, in our study, higher gut *α*-diversity in females compared to males was no longer observed in adults over the age of 45. A study by Flores et al. further demonstrated that the levels of urinary estrogen were associated with gut alpha-diversity in both males and postmenopausal females, but not in premenopausal women, highlighting the higher similarity between postmenopausal females and males (Flores et al., 2012). Another factor that could possibly play a role in the difference in diversity between younger and older women is birth control. It has been shown that birth control affects microbiota composition and diversity (Mihajlovic et al., 2021). As it is likely that most women over 45 years old do not take birth control anymore, its modulatory effect fades, which could help explain why the microbiota suffers alterations. It should be noted, however, that only 17 female participants (26% of females) reported being on birth control and that composition and dosage varied, making the possible effect on the microbiota unclear. Therefore, the menopausal-associated hormone changes seem to be the main explanatory factor for this difference. Interestingly, hormones might not have the same effect on the female oral microbiota, with a recent study reporting that the salivary microbiota of most women remained relatively stable throughout the menstrual cycle and in menopause (Tramice et al., 2022). Accordingly, our results also do not suggest menopausal-related alterations in the oral microbiota. Additionally, we did not detect sex-driven differences in the oral microbiota in any age group, whereas some studies in the literature report significant differences in oral *β*-diversity between adult males and females (Minty et al., 2021; Liu et al., 2023).

In addition to exploring the effect of age and sex, the relation between BMI and the microbiota was also evaluated. Gut microbiota differed significantly between overweight and normal-weight adults, with the normal-weight group exhibiting a higher abundance of 14 out of 16 genera. This was expected, considering that gut diversity is reported to decrease as BMI values increase, with the gut microbiota of overweight individuals being less diverse than that of people considered to have a normal-weight (Yun et al., 2017; Himbert et al., 2022). The same scenario is reported for the oral microbiota (Wu et al., 2018; Bu et al., 2019), although we did not observe significant changes between normal-weight and overweight individuals in this study. Additionally, overweight adults exhibited a higher prevalence of the genera *Neisseria* and *Sutterella*, both previously reported to be associated with obesity (Bombin et al., 2022; Pinart et al., 2022). Curiously, when it comes to underweight individuals, differences were detected in the oral microbiota but not in the gut microbiota, with the underweight group exhibiting a lower prevalence of all 31 genera identified. Although the oral microbiota in overweight individuals is typically associated with lower α-diversity, the underweight microbiota does not seem to significantly differ from that of normal-weight individuals (Bu et al., 2019). On the other hand, controversial results have been reported regarding whether gut diversity increases (Gao et al., 2018) or decreases (Wan et al., 2020) in underweight participants compared to normal-weight individuals. It is imperative to mention that our results regarding the underweight group should be viewed critically as only a total of four participants were classified as underweight. Additionally, only 27 participants qualified as overweight while obese participants (BMI ≥ 30 kg/m^2^) were excluded from the original cohort altogether, as the effect of BMI was not the focus of the study. Thus, a much larger number of participants should be involved in order to obtain significant results. Regarding children, although the literature reports differences between underweight, normal and overweight children, both regarding the oral and gut microbiota (Mervish et al., 2019; Burcham et al., 2020), no significant differences whatsoever were detected in this age group between normal, overweight or obese children, possibly because of the reduced number of participants in each group (24 children qualified as normal weight, while three children qualified as overweight, and four qualified as obese; information relating to eight children was missing). A significantly larger number of participants would be necessary to further explore this connection.

Other variables tested in this study (adult educational level and oral hygiene habits) do not seem to impact the results, which is unsurprising given the fact that our adult population is fairly homogenous. Interestingly, variables which demonstrated some level of impact on the results, like age, sex, and BMI, seem to condition the gut milieu much more than the oral cavity, consequently having a more measurable impact on the gut microbiota.

Aside from the ones already mentioned, this study presents some limitations: although we explored the oral-gut relationship in children and adults, a longitudinal study following the same individuals at different ages would allow for clearer conclusions regarding microbiota maturation. Including several timepoints would also clarify if the microorganisms translocating from the oral cavity to the gut are able to establish themselves in the gut and, if so, for how long. Thus, microbial viability should also be explored. However, the fact that from the subset of 61 ASVs shared between the oral cavity and gut of the same individual, 26 ASVs are also present in both children and adults is a good indicator that their presence in both habitats is likely not transitory and probably persists across age groups. These results highlight the likelihood of oral-to-gut translocation in healthy individuals, possibly established from an early age. Nevertheless, characterization of the microbiota on a strain level should be carried out in order to confirm translocation. Geographic location should also be considered when reporting results, as all participants in the study are Portuguese (Southern European), and cohorts from other geographic regions might present different sets of shared ASVs. Additionally, oral health and oral hygiene habits were only recorded for adults, which made it impossible to control for these factors in children.

## 5 Conclusion

Overall, although the oral and gut microbiota differ vastly, we were still able to identify a subset of 61 ASVs (representing 1.6% of all ASVs detected) present in both the oral cavity and gut of the same individual, with at least one shared ASV being detected in 96% of participants. From these, 26 ASVs (classified into 18 genera) were also present in both children and adults, suggesting that these microorganisms are likely persistent (and not just transitory) colonizers of both habitats, with shared colonization initiating in childhood. The fact that 62% of shared ASVs were more abundant in the oral cavity suggests oral-to-gut translocation as the main route of translocation between habitats, proposing that this phenomenon might be more common than previously thought in healthy individuals. Thus, shared oral-gut bacteria should be thoroughly studied in the future so that their role in both habitats and its influence on host health and wellbeing can be understood. Bacteria exclusively detected in the oral cavity should also be studied in order to understand if possible translocation would play a part in disease development. As for age, microbial composition differs greatly between children and adults, with large numbers of ASVs being exclusively detected in each age group, thus suggesting age-related maturation of the microbiota. Bacterial diversity was relatively stable between life stages, with significant changes being detected only after 45 years of age. Factors such as sex, age, and BMI seem to have a more significant impact on the gut microbiota than on the oral cavity.

## Data Availability

The datasets presented in this study can be found in online repositories. The names of the repository/repositories and accession number(s) can be found at: https://www.ncbi.nlm.nih.gov/, PRJNA1161112 and PRJNA1161042.
