# Supplementary material for: The oral-gut microbiota relationship in healthy humans: identifying shared bacteria between environments and age groups
Source: Front Microbiol. 2024 Oct 24;15:1475159. doi: 10.3389/fmicb.2024.1475159 (PMC11540997; doi:10.3389/fmicb.2024.1475159)
Supplement: Supplementary file 1 [file Data_Sheet_1.docx]

Supplementary Material

# DNA extraction protocols

## DNA extraction from stool samples

DNA extraction from stool samples was done utilising the DNeasy Blood & Tissue Kit (Qiagen, Germany), following a modified version of the protocol of the QIAamp DNA Stool Mini Kit (Qiagen, Germany) described in the QIAamp DNA Stool Handbook (Qiagen, 2010) available on the Qiagen website. All reagents were used as provided in the DNeasy Blood & Tissue Kit. InhibitEX Buffer (Qiagen, Germany) and ethanol for molecular biology (Merck, Germany) were purchased separately.

Protocol for stool DNA extraction:

Firstly, 180-220 mg of stool were weighed into Lysing Matrix D 2 mL tubes (MP Biomedicals, USA) and placed on ice; InhibitEX Buffer (Qiagen, Germany) was added to each sample; Samples were then homogenised using a FastPrep-24 (MP Biomedicals, USA) at 4.0 m/sec for 1 min; The resulting suspension was then transferred into new 1.5 mL microcentrifuge tubes (VWR International, LLC, USA), heated for 5 min at 95ºC in a ThermoMixer (Eppendorf, Germany), vortexed for 10 s and centrifuged at full speed for 1 min; 15 µL of proteinase K were pipetted into new 1.5 mL microcentrifuge tubes, followed by 200 µL of the supernatant resulting from the previous steps; 200 µL of Buffer AL were added and the mixture was vortexed for 10 s and then incubated for 10 min at 95ºC in a ThermoMixer; 200 µL of ethanol (96-100%) were added and the mixture was vortexed for 10 s; New DNeasy Mini spin columns were placed in 2 mL collection tubes (VWR International, LLC, USA) and 600 µL of the lysates were carefully transferred to the columns, which were then centrifuged at full speed for 1 min; The filtrate was discarded and 500 µL of Buffer AW1 were added to the spin columns, which were then centrifuged at full speed for 1 min; The filtrate was once again discarded and 500 µL of Buffer AW2 were added to the spin columns, which were then centrifuged at full speed for 3 min; After discarding the filtrate, in order to fully dry the membrane of the DNeasy Mini spin columns, these were centrifuged at full speed for an additional 3 min (without the addition of any reagents); Finally, the spin columns were transferred into new 1.5 mL microcentrifuge tubes and 100 µL of Buffer AE were pipetted directly into the column membrane; After incubation for 1 min at room temperature, the columns were centrifuged at full speed for 1 min to elute DNA. Final volume of DNA per sample = 100 µL.

## DNA extraction from saliva samples

DNA extraction from saliva samples was done utilising the DNeasy Blood & Tissue Kit (Qiagen, Germany), following a modified version of the “User-Developed Protocol: Purification of total DNA from animal saliva using the DNeasy® Blood & Tissue Kit” (Qiagen, 2006) available on the Qiagen website. All reagents were used as provided in the DNeasy Blood & Tissue Kit. Ethanol for molecular biology (Merck, Germany) was purchased separately.

Protocol for saliva DNA extraction:

Samples (stored at -80ºC) were allowed to thaw; As a quick thawing is preferred, samples were placed in an oven at 37ºC; 250 µL of saliva were transferred into new 1.5 mL microcentrifuge tubes and centrifuged at 12,000 rpm speed for 8 min; After carefully decanting the supernatant, the resulting pellets were resuspended in 180 µL Buffer ATL and vortexed for 15 s; The samples were then placed in an Ultrasonic Bath (Bandelin Electronic, Germany) for 3 min to further disrupt the biofilm and endure destruction of the cell wall (Hohnadel et al., 2014); The samples were then vortexed for an additional 30 s or until the pellets were fully dissolved; 25 µl of proteinase K were added and the mixture was vortexed for 10 s; After adding 200 µL of Buffer AL (without added ethanol) and vortexing for 15 s, the samples were incubated for 10 min at 56ºC in a ThermoMixer; 200 µL of ethanol (96-100%) were added and the mixture was vortexed for 15 s; New DNeasy Mini spin columns were placed in 2 mL collection tubes and the mixture was carefully transferred to the columns, which were then centrifuged at full speed for 1 min; The filtrate was discarded and 500 µL of Buffer AW1 were added to the spin columns, which were then centrifuged at full speed for 1 min; The filtrate was once again discarded and 500 µL of Buffer AW2 were added to the spin columns, which were then centrifuged at full speed for 3 min; After discarding the filtrate, in order to fully dry the membrane of the DNeasy Mini spin columns, these were centrifuged at full speed for an additional 3 min (without the addition of any reagents); Finally, the spin columns were transferred into new 1.5 mL microcentrifuge tubes and 30 µL of Buffer AE were pipetted directly into the column membrane; After incubation for 3 min at room temperature, the columns were centrifuged at 9,000 rpm for 1 min to elute DNA. For maximum DNA yield, the elution step was repeated (30 µL of Buffer AE were pipetted directly into the column membrane and after incubation for 3 min at room temperature, the columns were centrifuged at 9,000 rpm for 1 min to elute DNA into the same tube). Final volume of DNA per sample = 60 µL.

Reference:

Hohnadel, M., Felden, L., Fijuljanin, D., Jouette, S., Chollet, R. (2014). A new ultrasonic high-throughput instrument for rapid DNA release from microorganisms. Journal of Microbiological Methods 99, 71-80. doi: 10.1016/j.mimet.2014.02.004.

# Supplementary Figures


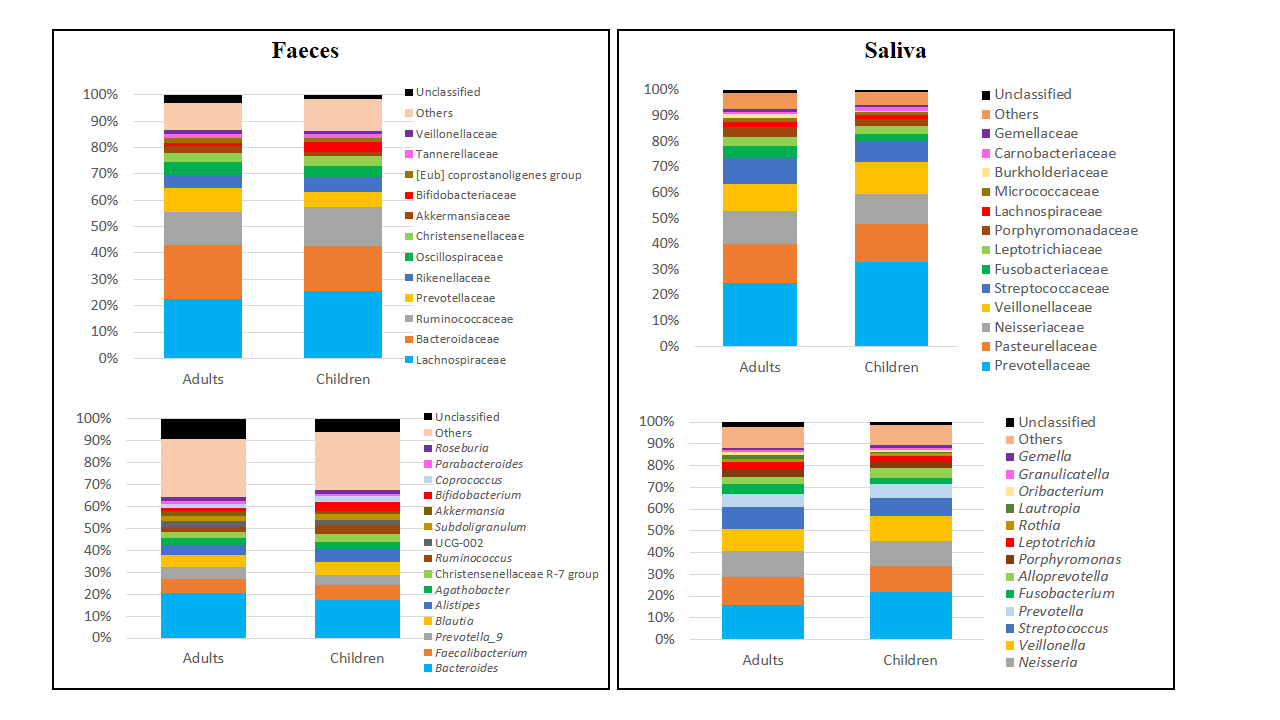


**Supplementary Figure 1.** The figure legends are required to have the same font as the main text, 12 point normal Times New Roman, single spaced. Please use a single paragraph for each legend and prepare the figures keeping in mind the PDF layout.

**Supplementary Figure 1.** Taxonomic classification (family and genus) and distribution of gut and oral samples in adults and children. The analyses were done in STAMP 2.1.3 (https://beikolab.cs.dal.ca/software/STAMP).


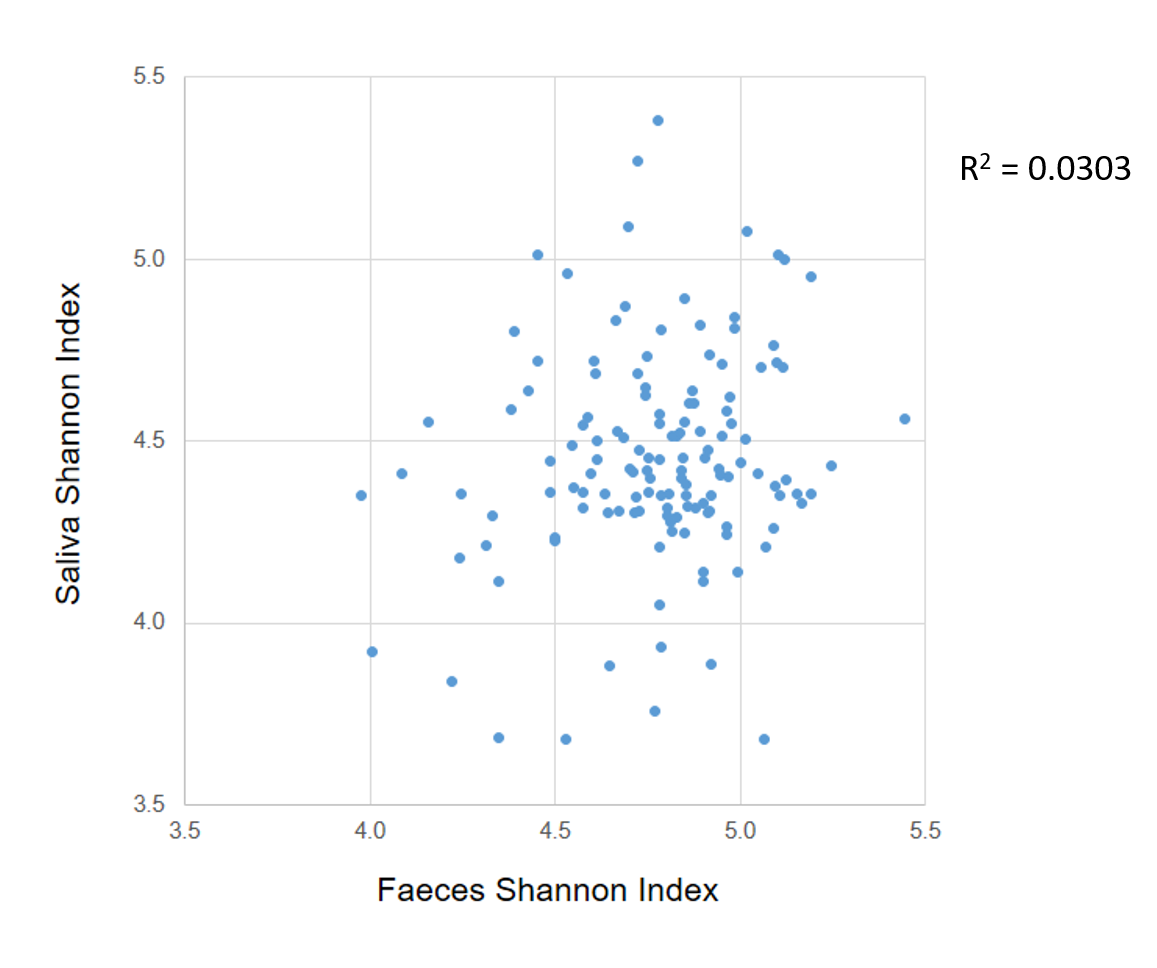


**Supplementary Figure 2.** Correlation between the Shannon diversity index of oral and faecal samples.


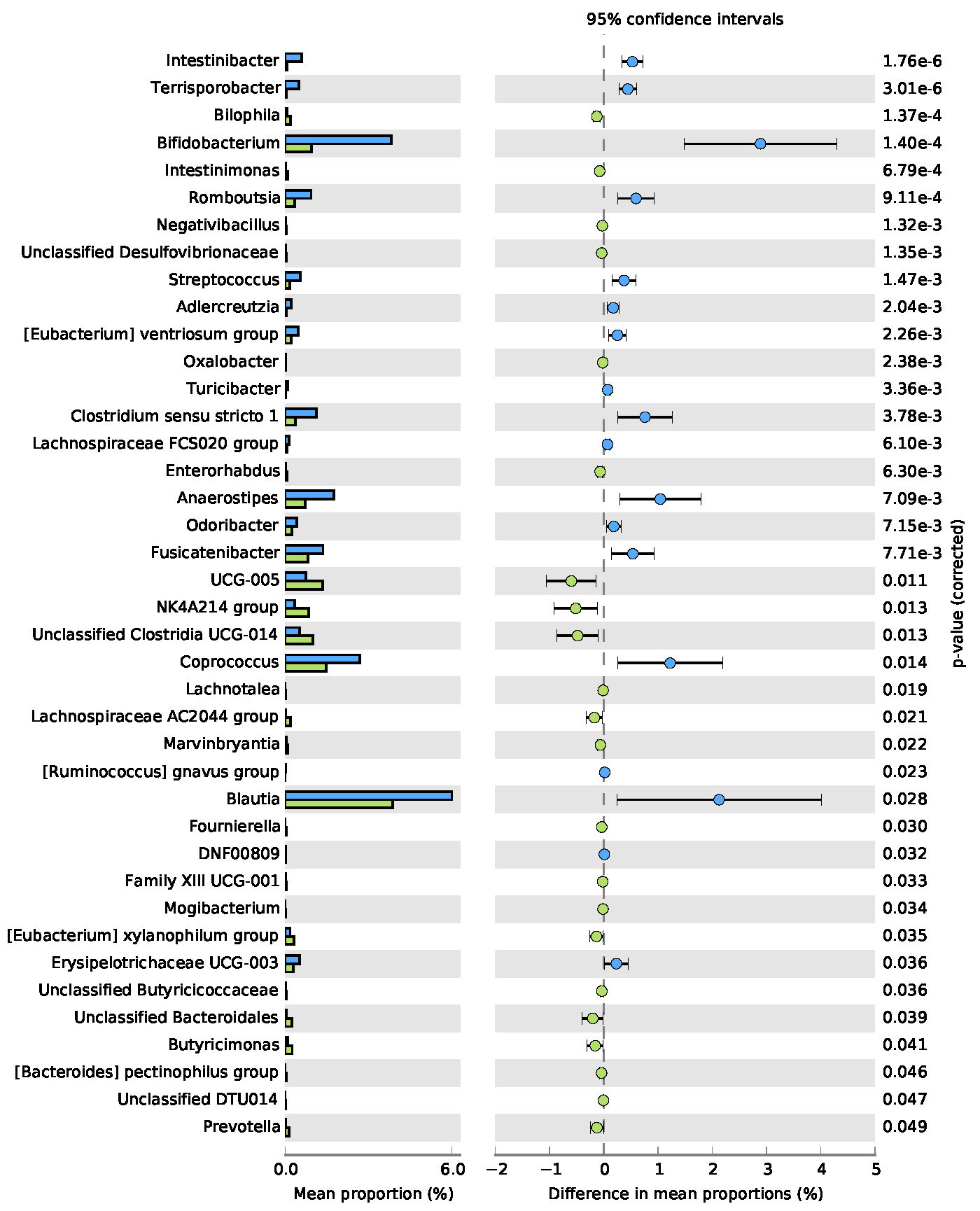


**Supplementary Figure 3.** Post-hoc analyses showing differences on taxonomic groups of gut microbiota between children (blue) versus older adults (age group over 45 years old; green). The analyses were done in STAMP 2.1.3 (https://beikolab.cs.dal.ca/software/STAMP).


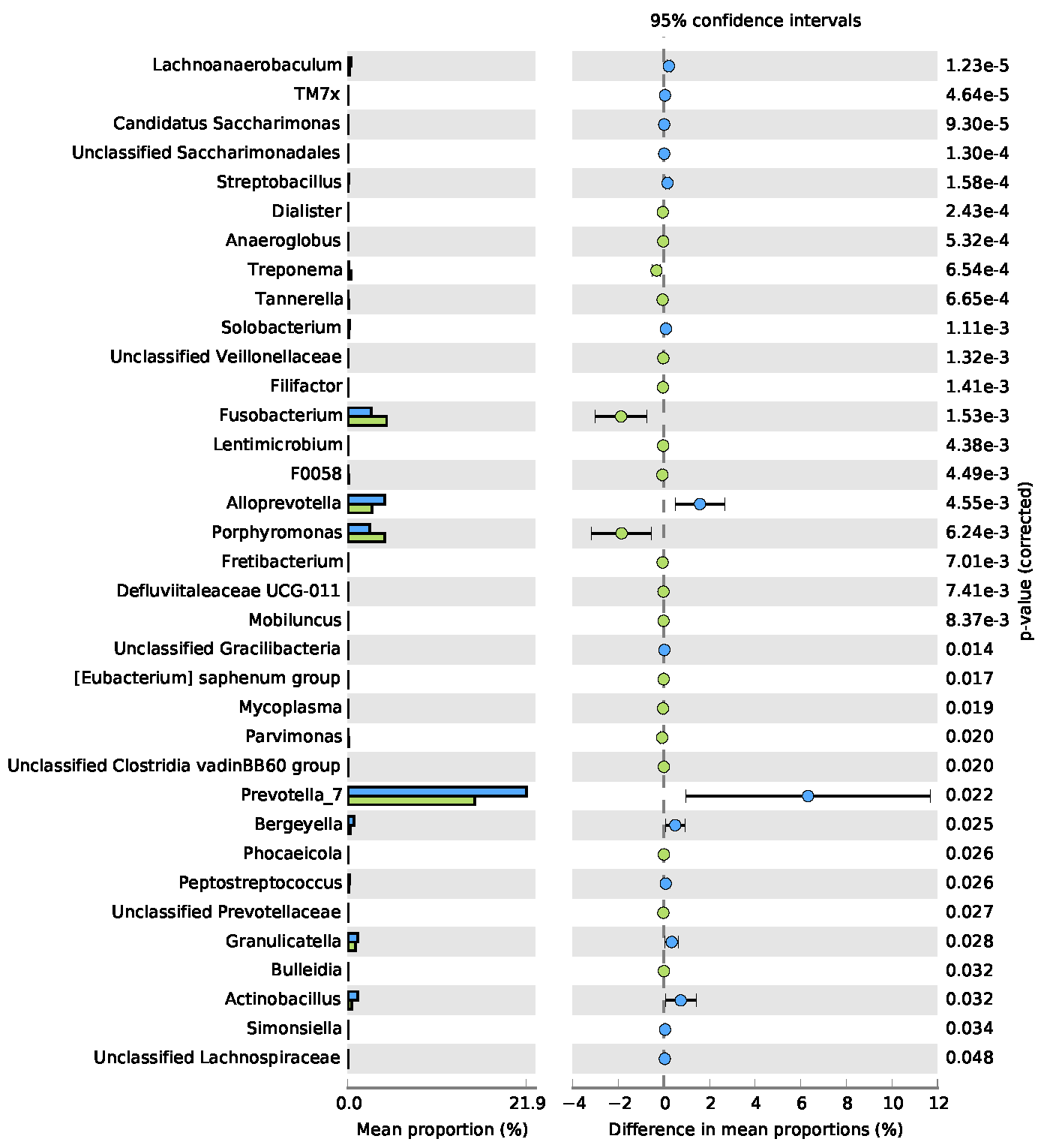


**Supplementary Figure 4.** Post-hoc analyses showing differences on taxonomic groups of oral microbiota between children (blue) versus older adults (age group over 45 years old; green). The analyses were done in STAMP 2.1.3 (https://beikolab.cs.dal.ca/software/STAMP).
